# Enhancement of CD4^+^ T Cell Function as a Strategy for Improving Antibiotic Therapy Efficacy in Tuberculosis: Does It Work?

**DOI:** 10.3389/fcimb.2021.672527

**Published:** 2021-06-21

**Authors:** Diego L. Costa, Eduardo P. Amaral, Sivaranjani Namasivayam, Lara R. Mittereder, Bruno B. Andrade, Alan Sher

**Affiliations:** ^1^ Departmento de Bioquímica e Imunologia, Faculdade de Medicina de Ribeirão Preto, Universidade de São Paulo, Ribeirão Preto, Brazil; ^2^ Programa de Pós-Graduação em Imunologia Básica e Aplicada, Faculdade de Medicina de Ribeirão Preto, Universidade de São Paulo, Ribeirão Preto, Brazil; ^3^ Immunobiology Section, Laboratory of Parasitic Diseases, National Institute of Allergy and Infectious Diseases, National Institutes of Health, Bethesda, MD, United States; ^4^ Division of Bacterial, Parasitic and Allergenic Products, Laboratory of Mucosal Pathogens and Cellular Immunology, Center for Biologics Evaluation and Research, Food and Drug Administration, Silver Spring, MD, United States; ^5^ Wellcome Centre for Infectious Disease Research in Africa, Institute of Infectious Disease and Molecular Medicine, University of Cape Town, Cape Town, South Africa; ^6^ Laboratório de Inflamação e Biomarcadores, Instituto Gonçalo Moniz, Fundação Oswaldo Cruz, Salvador, Brazil; ^7^ Multinational Organization Network Sponsoring Translational and Epidemiological Research (MONSTER) Initiative, Salvador, Brazil; ^8^ Curso de Medicina, Faculdade de Tecnologia e Ciências (FTC), Salvador, Brazil; ^9^ Curso de Medicina, Universidade Salvador (UNIFACS), Laureate Universities, Salvador, Brazil; ^10^ Escola Bahiana de Medicina e Saúde Pública (EBMSP), Salvador, Brazil; ^11^ Division of Infectious Diseases, Department of Medicine, Vanderbilt University School of Medicine, Nashville, TN, United States

**Keywords:** tuberculosis, host-directed therapy, adaptive immunity, CD4^+^ T lymphocytes, IFN-γ, TNF, IL-12

## Abstract

Tuberculosis (TB), caused by *Mycobacterium tuberculosis* (Mtb) remains a major public health problem worldwide due in part to the lack of an effective vaccine and to the lengthy course of antibiotic treatment required for successful cure. Combined immuno/chemotherapeutic intervention represents a major strategy for developing more effective therapies against this important pathogen. Because of the major role of CD4^+^ T cells in containing Mtb infection, augmentation of bacterial specific CD4^+^ T cell responses has been considered as an approach in achieving this aim. Here we present new data from our own research aimed at determining whether boosting CD4^+^ T cell responses can promote antibiotic clearance. In these studies, we first characterized the impact of antibiotic treatment of infected mice on Th1 responses to major Mtb antigens and then performed experiments aimed at sustaining CD4^+^ T cell responsiveness during antibiotic treatment. These included IL-12 infusion, immunization with ESAT-6 and Ag85B immunodominant peptides and adoptive transfer of Th1-polarized CD4^+^ T cells specific for ESAT-6 or Ag85B during the initial month of chemotherapy. These approaches failed to enhance antibiotic clearance of Mtb, indicating that boosting Th1 responses to immunogenic Mtb antigens highly expressed by actively dividing bacteria is not an effective strategy to be used in the initial phase of antibiotic treatment, perhaps because replicating organisms are the first to be eliminated by the drugs. These results are discussed in the context of previously published findings addressing this concept along with possible alternate approaches for harnessing Th1 immunity as an adjunct to chemotherapy.

## Introduction

Tuberculosis (TB), caused by the infection with the bacterium *Mycobacterium tuberculosis* (Mtb) is the most lethal infectious disease in human history and estimates indicate that it has been responsible for the death of nearly one billion people in the past two centuries alone (Paulson, 2013). In the present days, TB is still a major public health problem worldwide, with close to 10 million new cases and 1.4 million deaths (208,000 of which were due to Mtb-HIV co-infection) reported in 2019 (WHO, 2019).

The only currently licensed vaccine for TB is BCG (Bacillus Calmette–Guérin), which was first deployed in 1921 (Calmette, 1931). Clinical studies have failed to demonstrate its uniform efficacy, identifying variable protection rates that may range from as high as 80% to no protection at all (Aronson et al., 2004; Soysal et al., 2005; Tuberculosis Research, 2013). Antibiotic treatment for TB is available, however it consists of 4 different antibiotics administered for six to nine months in patients infected with drug-susceptible Mtb. In people infected with antibiotic-resistant bacteria, therapy can last up to 24 months, employs more toxic drugs and has a low success rate (WHO, 2019). Importantly, the side effects associated with prolonged treatment frequently lead to non-compliance which can promote disease reactivation and the emergence of drug resistant bacteria. These problems combined with the absence of a proven effective vaccine for the prevention of adult TB help explain why this disease has been so difficult to control let alone eradicate.

While the search for more effective antibiotics continues, a more recent focus has been on treatment strategies that target host biological processes identified as playing important roles in the disease pathogenesis, also known as host-directed therapies. Of these, a major approach is to boost protective immune responses against Mtb in conjunction with antibiotic therapy in order to accelerate pathogen clearance (Wallis and Hafner, 2015). Promising results have been obtained utilizing this general strategy. Many of these employ therapeutic administration of cytokines that are critical for host protection against Mtb infection, such as IFN-γ, produced mainly by CD4^+^ Th1 cells (Saravia et al., 2019), which resulted in improved control of bacterial replication and tissue damage in patients (Condos et al., 1997; Suarez-Mendez et al., 2004; Grahmann and Braun, 2008). Along the same lines, vaccination strategies known to potentiate host T cell responses against Mtb have been used as an adjunct to antibiotic therapy in Mtb-infected patients and animals, resulting in accelerated bacterial clearance (Skinner et al., 1997; Gupta et al., 2012; Coler et al., 2013; Huang and Hsieh, 2017; Sharma et al., 2017).

In this perspective article, we present new data from experiments in which we employ an Mtb mouse model and epitope specific flow cytometric analyses to re-examine different strategies for boosting CD4^+^ T cell responses to Mtb antigens concomitant with antibiotic therapy in Mtb-infected mice. We then discuss our findings in the light of those from the previous studies referred to above to suggest alternative approaches for improving the outcome of this interventional strategy.

## Material and Methods

### Mice, Experimental Infection, and Bacterial Load Quantification

C57BL/6, B6.SLJ, Rag1^-/-^ and TCRα^-/-^ mice were acquired through a National Institute of Allergy and Infectious Diseases (NIAID) supply contract with Taconic Farms (Germantown, NY), while C7 TCR P25 TCR mice were kindly provided by Dr. Michael Glickman from Sloan Kettering Memorial Cancer and Dr. Joel Ernst from University of California San Francisco, respectively. C7 TCR and P25 TCR mice were crossed with Rag1^-/-^ mice to generate C7 Rag1^-/-^ and P25 Rag1^-/-^ animals. Mice were housed at Biosafety levels 2 and 3 facilities at the NIAID, National Institutes of Health (NIH) and all the experimental protocols were approved by the NIAID Animal Care and Use Committee (ACUC). Mice were infected through aerosol route using a whole-body exposure/inhalation system (Glas Col, Terre Haute, IN) with an average of 100 CFU of *M. tuberculosis* (Mtb) strain H37Rv (inoculum was assessed for every exposure procedure and only those in which CFU values were between 50 to 150 were used in experiments). Bacterial loads were quantified in lung homogenates through limiting dilution in 7H11 agar medium (Sigma-Aldrich, Saint Louis, MO) enriched with OADC (BD Biosciences, San Jose, CA).

### Reagents and Treatment Regimens

Antibiotic treatment was initiated at 14 or 28 days post-infection. All other treatments were initiated at or after 28 days post-infection. Antibiotic administration (rifampicin, isoniazid and pyrazinamide - all from Sigma-Aldrich - at 10, 25 and 150 mg/kg respectively) was performed orally by gavage (5 days/week), as previously published (Costa et al., 2016). IL-12, kindly provided by Dr. Giorgio Trinchieri, National Cancer Institute, NIH). was intraperitoneally injected (350 ng of recombinant murine IL-12p70/mouse/dose). Mice treated with rmIL-12p70 had increased numbers of splenocytes compared to animals that were not treated with the cytokine (not shown), confirming its biological activity (splenomegaly is a common effect associated with IL-12p70 administration) (Car et al., 1995). ESAT-6_1-20_ (MTEQQWNFAGIEAAASAIQG) or Ag85B_280-294_ (FQDAYNAAGGHNAVF) peptides (100 μg/mouse/dose) were intravenously injected as were C7 or P25 TCR transgenic Th1-polarized CD4^+^ T cells (5 x 10^5^ cells/mouse/dose). *In vitro* Th1 polarization was performed as previously described (Sallin et al., 2017).

### Lung Processing, Quantification of IL-12p40 and Flow Cytometry

At different time points post-Mtb infection and treatment (days 28, 38, 48, 56, 58, 68, 78 and 88 post-infection, or alternatively, days 0, 10, 20, 28, 30, 40, 50 and 60 post-treatment, individually indicated in figure legends), mice were euthanized and lungs were perfused with sterile PBS, excised and processed. For cytokine quantification, the organs were disrupted in 2 ml tubes containing 2.7 mm glass beads in PBS with cOmplete ULTRA™ protease inhibitor cocktail (Roche, Basel Switzerland) or Trizol reagent (ThermoFisher Scientific, Waltham, MA) for Elisa and real time PCR respectively, using a Precellys Evolution™ tissue homogenizer (Bertin Instruments, Montigny-le-Bretonneux, France). Elisa for IL-12p40 (Becton & Dickinson, San Jose, CA) detection was performed according to manufacturer’s instructions. For real-time PCR, total mRNA was extracted using Qiagen RNeasy mini kits (Qiagen, Hilden, Germany) and 1 μg of RNA was reverse transcribed into cDNA using superscript II reverse transcriptase and random primers (ThermoFisher Scientific). SYBR Green and 7900HT Fast Real-Time PCR (ThermoFisher Scientific) were used in real time PCR reactions. Relative expression of Il12b gene in Mtb-infected mouse lungs was normalized to that of β-actin and further analyzed in relation to those of uninfected naïve mice lungs. Murine primers used: Actb F: 5’ AGC TGC GTT TTA CAC CCT TT 3’; Actb R: 5’ AAG CCA TGC CAA TGT TGT CT 3’; Il12b F: 5’ AGC ACC AGC TTC TTC ATC AGG 3’; Il12b R: 5’ GCG CTG GAT TCG AAC AAA G 3’. For flow cytometry, lung single cell suspensions were prepared as previously described (Costa et al., 2020). For intracellular cytokine detection, prior to staining cells were incubated for 5 hours at 37°C with 10 µg/ml ESAT-6 _1-20_ or Ag85B _280-294_ in the presence of brefeldin A and monensin during the whole 5h culture period, as previously published (Sallin et al., 2018). Antibody clones used were: TCRβ (H57-597), CD4 (GK1.5), CD44 (IM7), Foxp3 (FJK-16s), IFNγ (XMG1.2), TNF (MP6-XT22) and Fixable Viability Dye eFluor 780 (Biolegend, San Jose, CA, BD Biosciences and ThermoFisher Scientific). Fluorochrome-labeled I-Ab ESAT-6 _1-20_ and I-Ab Ag85B _280-294_ tetramers were obtained from the NIAID tetramer facility (Atlanta, GA). Samples were acquired on a LSRII Fortessa cytometer (BD Biosciences) and analyzed using FlowJo software (FlowJo LLC, Ashland, OR).

### Statistical Analysis

Differences between groups were statistically assessed by unpaired Student’s t test using Prism software (GraphPad) and considered significant when *p ≤* 0.05.

## Results and Discussion

### Antibiotic Treatment Induces a Rapid Reduction in Pulmonary IL-12p40 Expression and Alters CD4^+^ T Cell Response to ESAT-6 and Ag85B

**Figure 1:**
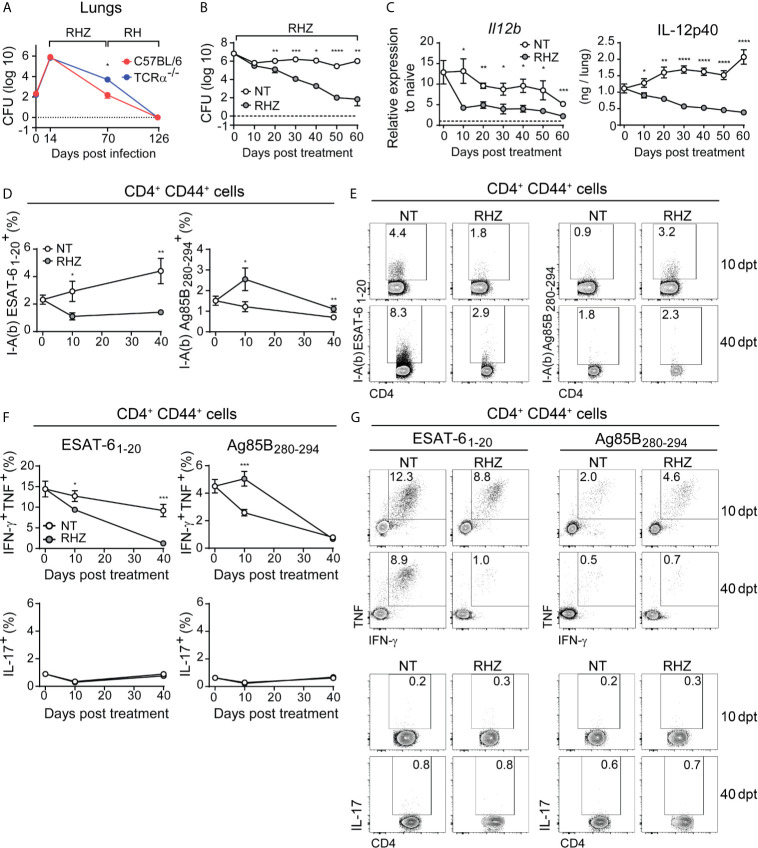
Antibiotic treatment of Mtb-infected mice results in rapid decrease in pulmonary IL-12p40 production and ESAT-6-specific Th1 response. **(A)** C57BL/6 (red dots and line) or TCRα^-/-^ (blue dots and line) mice were infected with 100 CFU of *M. tuberculosis* strain H37Rv and then treated, starting at 14 days post-infection (dpi), with a cocktail containing rifampicin (10 mg/kg), isoniazid (25 mg/kg) and pyrazinamide (150 mg/kg) (RHZ) for the first 56 days, followed by a continuation phase in which mice received only rifampicin (10 mg/kg), isoniazid (25 mg/kg) until 126 dpi. Graph shows pulmonary bacterial loads 70 dpi (56 days post-treatment - dpt) and 126 dpi (112 dpt); **(B–G)** C57BL/6 mice were infected with 100 CFU of *M. tuberculosis* strain H37Rv and then treated or not, starting at 28 dpi (day 0 post-treatment), with a cocktail containing rifampicin (10 mg/kg), isoniazid (25 mg/kg) and pyrazinamide (150 mg/kg) (RHZ); **(B)** Pulmonary bacterial loads in non-treated (NT – white dots) and antibiotic treated (RHZ – gray dots) Mtb-infected mice at 0, 10, 20, 30, 40, 50 and 60 dpt; **(C)** IL-12p40 mRNA expression (left panel) and protein quantification (right panel) in lung homogenates of non-treated (NT – white dots) or antibiotic treated (RHZ – gray dots) Mtb-infected mice at 0, 10, 20, 30, 40, 50 and 60 dpt; **(D)** Graphs and **(E)** representative dot plots depicting frequencies of ESAT-6 and Ag85B - specific CD4^+^CD44^+^ T lymphocytes (Singlets/Live/TCRβ^+^CD4^+^/Foxp3^-^/CD44^+^/tetramer^+^) in lungs of NT or RHZ treated Mtb-infected mice, 10 and 40 dpt; **(F)** Graphs and **(G)** representative dot plots depicting IFNγ and TNF double producing and IL-17-producing pulmonary CD4^+^CD44^+^ T lymphocytes (Singlets/Live/TCRβ^+^CD4^+^/Foxp3^-^/CD44^+^/IFNγ^+^TNF^+^or IL-17^+^) after *ex vivo* stimulation with ESAT-6 or Ag85B peptides in NT or RHZ treated Mtb-infected mice, at 10 and 40 dpt. Representative data of 2 independent experiments containing 4-5 mice/group are shown. Results are expressed as mean ± standard error of mean **(A–D, F)** and dot plots from flow cytometry concatenated data **(E, G)**. Statistical analysis: unpaired Student’s t test. *p ≤ 0.05, **p ≤ 0.01, ***p ≤ 0.001, ****p ≤ 0.0001.

T cell responses are critical for the containment of Mtb infection (Cooper, 2009) and may also play an important role in the effects of antibiotic treatment to TB. Patients that have TB-HIV co-infection present higher rates of antibiotic therapy failure and relapse compared to immunocompetent individuals with TB (Nahid et al., 2007; Bisson et al., 2015). In addition, using a murine model of high dose Mtb infection, Zhang et al. found that nude mice, which lack T lymphocytes, fail to clear Mtb infection when treated with antibiotics (Zhang et al., 2011). We have also tested whether T cells play a role in antibiotic treatment in experimental TB, but utilized a low-dose (~100 CFU) aerosol infection with Mtb strain H37Rv in C57BL/6 or TCRα^-/-^ mice. Treatment was initiated at 14 days post-infection (dpi), a time point at which no difference in pulmonary bacterial loads could be detected between C57BL/6 and TCRα^-/-^ mice (**Figure 1A**). Although we were unable to detect bacteria in the lungs of mice from both groups after 16 weeks of treatment (126 dpi) (**Figure 1A**), at an earlier phase, 8 weeks (70 dpi) post-treatment initiation, the pulmonary bacterial loads were higher in TCRα^-/-^ mice than C57BL/6 animals (**Figure 1A**). These results indicate that T cell-deficient mice display a delayed response to treatment and suggest therefore that concomitant induction of T cell responses might enhance the efficacy of antibiotic therapy in its initial phase.

Previous studies have shown that successful antibiotic treatment of TB patients results in decreased expression of pro-inflammatory cytokines (Cliff et al., 2015) as well as diminished T cell responses (Veenstra et al., 2007; Mattos et al., 2010). In order to evaluate the impact of antibiotic treatment on the production of Th1-associated cytokines in the lungs of Mtb-infected mice, we infected C57BL/6 animals with Mtb and treated them with antibiotics starting 28 dpi (day 0 of treatment). As expected, chemotherapy resulted in progressive reduction of pulmonary bacterial loads, which were significantly lower in treated animals compared to untreated mice at 20, 30, 40, 50 and 60 days post-treatment (**Figure 1B**). We also observed significantly lower IL-12p40 mRNA and protein levels in lungs of antibiotic-treated mice compared to untreated animals in all time points post-treatment (**Figure 1C**).

We next evaluated the pulmonary CD4^+^ T cell responses to two major immunodominant Mtb protein antigens; the first from ESAT-6, which is actively secreted by the bacilli (Pagan and Ramakrishnan, 2014), and the second from Ag85B, involved in the synthesis of the bacterial cell wall (Kremer et al., 2002). First, we used ESAT-6 or Ag85B peptide-MHC class II tetramers conjugated to fluorochromes to quantify antigen-specific activated (CD44^+^) CD4^+^ T cells in the lungs of Mtb-infected mice. Antibiotic treatment resulted in a reduction of ESAT-6-specific CD4^+^ T lymphocytes and an increase in Ag85B-specific cells at both 10 and 40 days post-treatment (**Figures 1D, E**). We also quantified the frequency of IFN-γ and TNF double-producer or IL-17 producer activated (CD44^+^) CD4^+^ T lymphocytes among leukocytes isolated from Mtb-infected mouse lungs after *ex vivo* stimulation with ESAT-6 or Ag85B peptides. The frequencies of IFN-γ and TNF double-positive CD4^+^ T cells following ESAT-6 stimulation were lower in samples of antibiotic-treated mice compared to those of untreated animals, at both 10 and 40 days post-treatment time points (**Figures 1F, G**). In contrast, Ag85B stimulation resulted in higher frequency of IFN-γ and TNF double producer CD4^+^ T cells in samples of antibiotic treated mice compared to untreated animals at 10 days post-treatment, while no difference was observed at the day 40 (**Figures 1F, G**). IL-17 expression following ESAT-6 or Ag85B stimulation was similar in untreated and antibiotic-treated animals at both 10 and 40 days post-treatment (**Figures 1F, G**).

Together these results demonstrated that antibiotic treatment results in a rapidly initiated and progressive reduction in the pulmonary levels of IL-12p40 and in IFN-γ and TNF production by CD4^+^ T lymphocytes in response to ESAT-6. Interestingly, this reduction in cytokine production was evident as early as 10 days post-treatment initiation, a time point in which there was no significant difference in pulmonary bacterial loads (**Figure 1B**). A study by Tousif et al. found that antibiotic treatment of Mtb-infected mice results in decreased numbers of activated CD4^+^ T cells, which was found to be caused by isoniazid-induced apoptosis of activated CD4^+^ T lymphocytes (Tousif et al., 2014). However, these findings contrast with the increase in Ag85B- (as opposed to ESAT-6) specific CD4^+^ T cells found in antibiotic-treated Mtb-infected mice (**Figures 1D, E**). Prior studies have shown that antibiotic (isoniazid) treatment upregulates the expression of proteins of the Ag85 complex *in vitro* (Garbe et al., 1996). Thus, it is possible that the post-treatment increases in Ag85B-specific CD4^+^ T cell levels observed in our experiments may be the consequence of an isoniazid-induced increase in the bacterial expression of Ag85 complex proteins in the mice receiving antibiotics. However, this initial enhancement of IFN-γ and TNF production in response to this antigen subsides at later time points, resulting in equally low responsiveness to Ag85B in treated and non-treated mice, which was previously demonstrated, in the case of untreated animals, to occur due to the natural reduction in the expression of this antigen by the bacteria in chronic phases of infection (Moguche et al., 2017; Ernst et al., 2019). In fact, the expression of a number of Mtb genes changes in response to stress conditions such as those triggered by antibiotic treatment (Briffotaux et al., 2019). Therefore, we favor the hypothesis that the early effects of antibiotic therapy on antigen specific CD4^+^ T cell responses results from alterations in bacterial gene and protein expression in response to antibiotic-induced stress rather than to apoptosis of CD4^+^ T cells.

In humans, successful treatment was also shown to result in reduction of IFN-γ levels in bronchoalveolar lavage fluid (BAL), although the antigen-specificity of this response was not assessed (Tsao et al., 2002). In peripheral blood mononuclear cells antibiotic therapy induced reduction of IFN-γ secretion by CD4^+^ T lymphocytes in response to ESAT-6 peptides and Ag85 (Pathan et al., 2001; Feruglio et al., 2015) measured at 4 and 2 weeks post-treatment, respectively. Our results regarding ESAT-6 responses are consistent with these findings, but differ from those involving Ag85.

Regardless, our findings establish that drug therapy results in a parallel decline in both IL-12 and ESAT-6-specific T cells producing Th1 cytokines, while Ag85B-specific Th1 responses initially increase in response to treatment, but will also decrease at later time points. Since both IL-12 and Th1 responses have been shown to be important for host control of infection and undergo dramatic changes in the initial phase of antibiotic treatment, we next asked whether boosting and sustaining them during the first month of therapy would improve treatment outcome.

### Strategies Aimed at Boosting Th1 Responses to Ag85B and ESAT-6 Antigens Fail to Accelerate Bacterial Clearance During the First Month of Antibiotic Treatment

**Figure 2:**
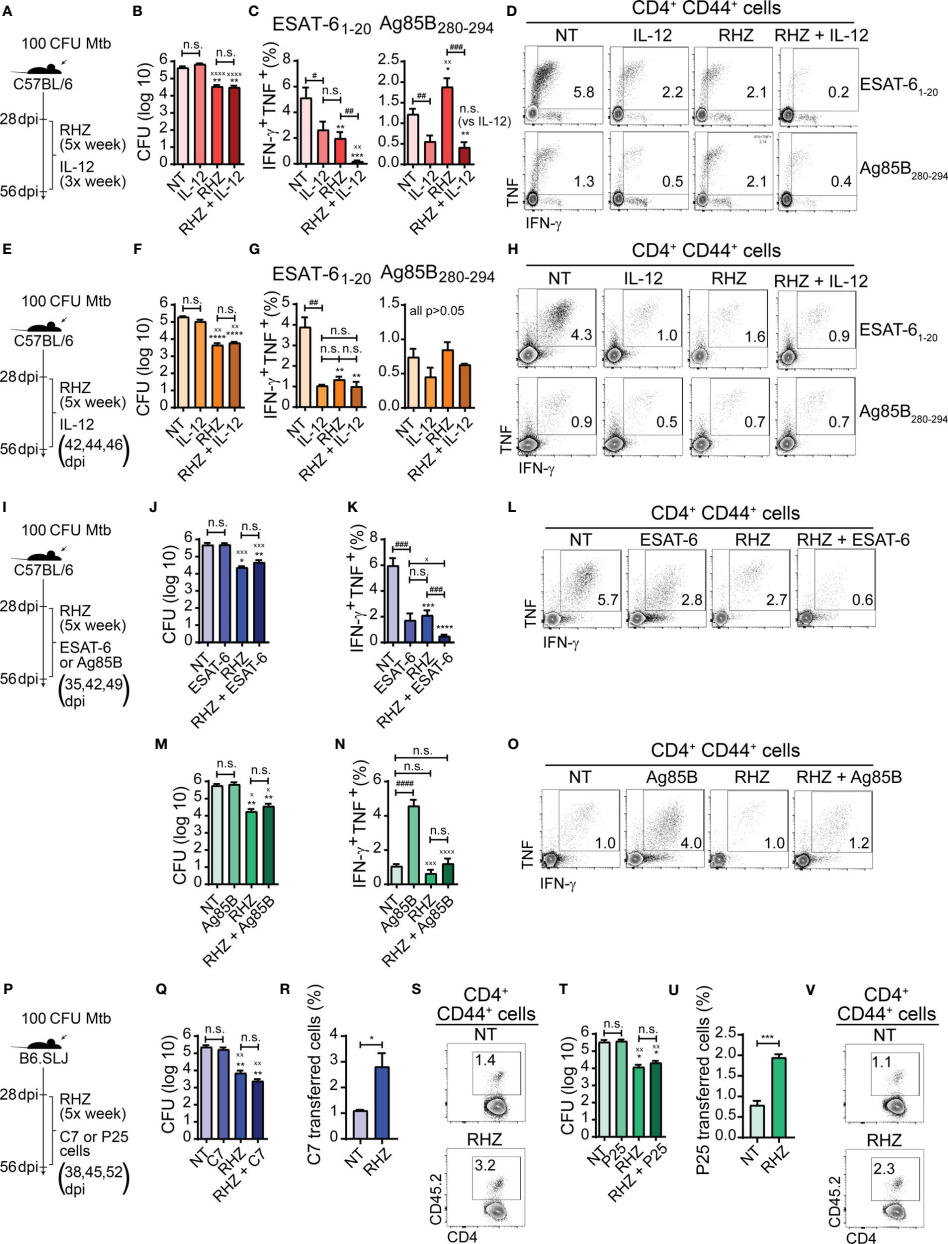
Strategies focused on enhancement of Th1 responses fail to improve bacterial clearance in Mtb-infected mice during the first four weeks of antibiotic treatment. In all experiments, C57BL/6 or B6.SLJ mice were treated or not, starting at 28 days post-infection (dpi) with a cocktail containing rifampicin (10 mg/kg), isoniazid (25 mg/kg) and pyrazinamide (150 mg/kg) (RHZ) for 28 days (until 56 dpi). **(A)** Treatment strategy for **(B–D)**, in which Mtb-infected mice were treated or not with RHZ in combination or not with recombinant murine IL-12p70 (350 ng/mouse/dose, 3× per week - between 28 and 56 dpi - intraperitoneally); **(B)** Pulmonary bacterial loads in Mtb-infected mice treated as in described in **(A)**, at 56 dpi (28 days post-treatment - dpt); **(C)** Graphs and **(D)** representative dot plots of IFNγ and TNF-producing pulmonary CD4^+^CD44^+^ T lymphocytes (Singlets/Live/TCRβ^+^CD4^+^/Foxp3^-^/CD44^+^/IFNγ^+^TNF^+^) after *ex vivo* stimulation with ESAT-6 or Ag85B peptide, which were obtained from Mtb-infected mice treated as in described in **(A)** at 56 dpi (28 dpt); **(E)** Treatment strategy for **(F-H)**, in which Mtb-infected mice were treated or not with RHZ in combination or not with recombinant murine IL-12p70 (350 ng/mouse/dose at 42, 44 and 46 dpi intraperitoneally); **(F)** Pulmonary bacterial loads in Mtb-infected mice treated as in described in **(E)**, at 56 dpi (28 dpt); **(G)** Graphs and **(H)** representative dot plots of IFNγ and TNF-producing pulmonary CD4^+^CD44^+^ T lymphocytes (Singlets/Live/TCRβ^+^CD4^+^/Foxp3^-^/CD44^+^/IFNγ^+^TNF^+^) after *ex vivo* stimulation with ESAT-6 or Ag85B peptide, which were obtained from Mtb-infected mice treated as in described in **(E)**, at 56 dpi (28 dpt); **(I)** Treatment strategy for **(J–O)**, in which Mtb-infected mice were treated or not with RHZ in combination or not with ESAT-6 _1-20_ peptide **(J–L)** (100 μg/mouse/dose at 35, 42 and 49 dpi, intravenously) or Ag85B 280-294 peptide **(M–O)** (100 μg/mouse/dose 35, 42 and 49 dpi, intravenously); **(J)** Pulmonary bacterial loads in Mtb-infected mice treated or not with RHZ in combination or not with ESAT-6 as in described in **(I)**, at 56 dpi (28 dpt); **(K)** Graphs and **(L)** representative dot plots of IFNγ and TNF-producing pulmonary CD4^+^CD44^+^ T lymphocytes (Singlets/Live/TCRβ^+^CD4^+^/Foxp3^-^/CD44^+^/IFNγ^+^TNF^+^) after *ex vivo* stimulation with ESAT-6 peptide, which were obtained from Mtb-infected mice treated or not with RHZ in combination or not with ESAT-6 as in described in **(I)**, at 56 dpi (28 dpt); **(M)** Pulmonary bacterial loads in Mtb-infected mice treated or not with RHZ in combination or not with Ag85B as in described in **(I)**, at 56 dpi (28 dpt); **(N)** Graphs and **(O)** representative dot plots of IFNγ and TNF-producing pulmonary CD4^+^CD44^+^ T lymphocytes (Singlets/Live/TCRβ^+^CD4^+^/Foxp3^-^/CD44^+^/IFNγ^+^TNF^+^) after *ex vivo* stimulation with Ag85B peptide, which were obtained from Mtb-infected mice treated or not with RHZ in combination or not with Ag85B as in described in **(I)**, at 56 dpi (28 dpt); **(P)** Treatment strategy for **(Q-V)**, in which Mtb-infected mice were treated or not with RHZ in combination or not with adoptive transfer of Th1-polarized C7 TCR transgenic CD4^+^ T cells **(Q–S)** (5 x 10^5^ cells/dose/mouse at 38, 45 and 52 dpi, intravenously) or Th1-polarized P25 TCR transgenic CD4^+^ T cells **(T–V)** (5 x 10^5^ cells/dose/mouse at 38, 45 and 52 dpi, intravenously); **(Q)** Pulmonary bacterial loads in Mtb-infected mice treated or not with RHZ in combination or not with C7 cell transfer as in described in **(P)**, at 56 dpi (28 dpt); **(R)** Graph and **(S)** representative dot plots of C7 TCR transferred cells (gated among Singlets/Live/TCRβ^+^CD4^+^FoxP3^-^CD44^+^ cells) isolated from lungs of Mtb-infected mice treated or not with RHZ in combination or not with C7 cell transfer as described in **(P)**, at 56 dpi (28 dpt); **(T)** Pulmonary bacterial loads in Mtb-infected mice treated or not with RHZ in combination or not with P25 cell transfer as in described in **(P)**, at 56 dpi (28 dpt); **(U)** Graph and **(V)** representative dot plots of P25 TCR transferred cells (gated among Singlets/Live/TCRβ^+^CD4^+^FoxP3^-^CD44^+^ cells) isolated from lungs of Mtb-infected mice treated or not with RHZ in combination or not with P25 cell transfer as in described in **(P)**, at 56 dpi (28 dpt). Representative data from a single experiment **(B–D, F–H, T–V)** or 2 independent experiments **(J–O, Q–S)**, all containing 4 mice/group are shown. Results are expressed as mean ± standard error of mean **(B, C, F, G, J, K, M, N, Q, R, T, U)** and dot plots from flow cytometry concatenated data **(D, H, L, O, S, V)**. Statistical analysis: unpaired Student’s t test. **(B, C, F, G, J, K, M, N, Q, T)** *p ≤ 0.05, **p ≤ 0.01, ***p ≤ 0.001 and ****p ≤ 0.0001 compared to NT; ^x^p ≤ 0.05, ^xx^p≤ 0.01, ^xxx^p≤ 0.001 and ^xxxx^p≤ 0.0001 compared to IL-12 or ESAT-6 or C7, or Ag85B or P25; ^#^p ≤ 0.05, ^##^p ≤ 0.01, ^###^p ≤ 0.001; ^####^p ≤ 0.0001 and n.s., non-significant, where indicated. **(R, U):** *p ≤ 0.05 and ***p ≤ 0.001.

IL-12 is composed of two subunits, IL-12p40 and IL-12p35, and its production is critical for host resistance to Mtb infection due to its crucial role in promoting the development of protective Th1 immunity (Feng et al., 2005). Genetic deletion of IL-12p40 subunit in mice results in impaired generation of Th1 adaptative immune responses, culminating in increased susceptibility to infection with Mtb (Cooper et al., 2002), and humans who carry mutations in IL12B (the IL-12p40 subunit) are highly susceptible to mycobacterial infections (Filipe-Santos et al., 2006). Elías-López et al. found that oral administration of extracts of transgenic tomatoes expressing recombinant murine IL-12 resulted in increased levels of the cytokine in the circulation and bronchoalveolar space of Mtb-infected mice, consequently improving the control of bacterial replication by the animals (Elias-Lopez et al., 2008). Similarly, Mata-Espinosa et al. demonstrated that intranasal administration of a genetically engineered adenoviral vector expressing mouse IL-12 during Mtb infection resulted in a significant reduction in pulmonary bacterial loads (Mata-Espinosa et al., 2019). However, the effects of IL-12 supplementation during antibiotic treatment of Mtb-infected mice to the best of our knowledge have never been investigated. Considering the importance of this cytokine for resistance to infection and the rapid and persistent reduction in IL-12p40 expression in pulmonary tissues of antibiotic-treated Mtb-infected mice, we wondered whether *in vivo* supplementation with IL-12 could accelerate the clearance of Mtb in pulmonary tissue by antibiotic therapy. Initially we tested a 4-week treatment strategy (from 28 to 56 dpi) in which Mtb-infected mice treated or not with antibiotics were injected with IL-12 intraperitoneally three times per week (**Figure 2A**). No decrease in bacterial loads was observed in mice treated with IL-12 alone compared to untreated animals and the concomitant administration of IL-12 and antibiotics did not induce further reductions in bacterial loads compared with that resulting from antibiotic treatment alone, although both antibiotic treated groups (RHZ and RHZ + IL-12) displayed lower bacillary burdens than untreated mice or IL-12 treated animals (**Figure 2B**). Unexpectedly, there was a lower frequency of IFN-γ and TNF double producing pulmonary CD4^+^ T cells after *ex vivo* stimulation with ESAT-6 and Ag85B peptides in lung cells from IL-12 injected mice compared to untreated animals (**Figures 2C, D**). As predicted, cells from antibiotic-treated mice displayed a decreased frequency of IFN-γ^+^ TNF^+^ CD4^+^ T cells in response to ESAT-6 stimulation compared to those of untreated animals, and these responses were not enhanced in IL- 12-treated mice. However, the percentages of IFN-γ and TNF double producing CD4^+^ T cells in response to ESAT-6 stimulation were further reduced in lungs of mice treated with antibiotics + IL-12 and were lower than the values obtained from samples from of all of the other groups (**Figures 2C, D**). Furthermore, the frequency of IFN-γ^+^ TNF^+^ CD4^+^ T lymphocytes after *ex vivo* Ag85B stimulation was higher in cells from antibiotic-treated mice compared to those of untreated and IL-12-treated animals. In addition, treatment with antibiotics + IL-12 resulted in a lower frequency of IFN-γ^+^ TNF^+^ CD4^+^ T cells in response to Ag85B peptide stimulation compared to samples from untreated and antibiotic-treated mice, but similar to those of mice treated with IL-12 only (**Figures 2C, D**). Transient lymphopenia is observed following IL-12 administration (Robertson et al., 1999) and we wondered if this effect might have impaired the recruitment of Th1 CD4^+^ T cells to mouse lungs following uninterrupted cytokine injections. Therefore, we repeated the experiment this time administering IL-12 only during the third week of treatment (42, 44 and 46 dpi) and allowing one week before euthanizing the mice (**Figure 2E**). Again, the pulmonary bacterial loads of untreated and IL-12 treated mice were similar, while animals treated with antibiotics or antibiotics + IL-12 displayed lower numbers of bacilli compared to untreated and IL-12 treated mice, and as in the prior experiment there was no difference in bacterial loads between antibiotics or antibiotics + IL-12 -treated mice (**Figure 2F**). Once again, the frequency of pulmonary IFN-γ and TNF-producing CD4^+^ T cells after *ex vivo* stimulation with ESAT-6 peptide was lower in the IL-12, antibiotics or antibiotics + IL-12 -treated animals compared with untreated mice, but was indistinguishable among the three first groups (**Figures 2G, H**). When pulmonary leukocytes were *ex vivo* stimulated with Ag85B peptide, the frequency of IFN-γ^+^ TNF^+^ CD4^+^ T cells was similar in all groups (**Figures 2G, H**).

The above findings indicated that while the levels of IL-12 decrease as a result of antibiotic treatment, co-administration of the cytokine does not sustain or increase the magnitude of pulmonary Th1 responses to the major immunodominant Mtb antigens ESAT-6 and Ag85B in immunocompetent Mtb-infected mice treated with antibiotics and may, on the contrary, induce their contraction. These results do not exclude the possibility that IL-12 infusion may have enhanced Th1 responsiveness to other Mtb antigens apart from ESAT-6 and Ag85. Nonetheless, the data demonstrate that IL-12 supplementation does not improve the control of Mtb infection by immunocompetent hosts nor accelerate the clearance of pathogens in the initial month of therapy when performed in conjunction with antibiotic treatment.

There are important side effects resulting from IL-12 supplementation that limit the use of direct cytokine administration, such as anemia, liver toxicity, splenomegaly, bone marrow hyperplasia, and lymphopenia (Gately et al., 1994; Sarmiento et al., 1994). The latter side effect may help explain the reduced levels of ESAT-6 and Ag85B-specific IFN-γ and TNF production by CD4^+^ T cells that we observed in animals treated with recombinant IL-12 for 3 weeks (**Figures 2C, D**). Additionally, ESAT-6 specific CD4^+^ T cells develop a terminally differentiated effector phenotype, while those specific to Ag85B acquire characteristics that resemble those of memory cells (Moguche et al., 2017). The one-week IL-12 infusion strategy used by us resulted in a drastic reduction of ESAT-6 responsive cells, while Ag85B responsiveness was unaltered (**Figures 2G, H**). Therefore, it is possible that terminally differentiated CD4^+^ T cells may be more susceptible to the detrimental effects of IL-12.

The production of IFN-γ and TNF are major players in Th1 immune response-mediated protection against Mtb infection (O’Garra et al., 2013). Since we observed that antibiotic therapy induced a rapid and persistent reduction in the production of IFN-γ and TNF by CD4^+^ T cells in response to ESAT-6 (**Figures 1F, G**), we decided to test whether sustaining or even enhancing ESAT-6 specific Th1 cell responsiveness in the lungs of antibiotic-treated animals might consequently result in more efficient bacterial clearance. Intravenous infusion of peptides was previously shown to result in stimulation of pulmonary peptide-specific CD4^+^ T cells *in vivo* in Mtb-infected mice (Moguche et al., 2017), and, therefore, we used the same approach to target ESAT-6-specific lung CD4^+^ T cells, administering or not ESAT-6_1-20_ peptide to untread or antibiotic-treated mice on days 7, 14 and 21 post-treatment (35, 42 and 49 dpi) (**Figure 2I**). We observed that pulmonary bacterial loads of ESAT-6-treated animals were similar to those of untreated mice. Animals treated with antibiotics or antibiotics + ESAT-6 also presented similar pulmonary bacterial loads, although lower than those from untreated or mice treated with ESAT-6 alone (**Figure 2J**). Interestingly, when we performed *ex vivo* stimulation of pulmonary leucocytes with ESAT-6 peptide, the frequency of IFN-γ^+^ TNF^+^ CD4^+^ T cells was lower in ESAT-6-treated mice compared to untreated animals but similar to antibiotic-treated mice. Therapy with antibiotics + ESAT-6 resulted in a further reduction in the frequency of IFN-γ^+^ TNF^+^ CD4^+^ T lymphocytes, which was lower in magnitude in mice that received this regimen compared to those of all of the other groups (**Figures 2K, L**). The above findings indicated that under the conditions employed by us, ESAT-6 supplementation fails to sustain or enhance Th1 responsiveness to this antigen and on the contrary, induces a contraction of this response in both groups of Mtb-infected mice whether treated or not with antibiotics. Additionally, ESAT-6 peptide co-administration did not improve the efficacy of antibiotic therapy.

The reduction in the frequency of ESAT-6-specific CD4^+^ T lymphocytes following peptide administration was surprising, considering that this approach was intended to sustain or enhance the pulmonary Th1 responses to this antigen. However, as mentioned previously, during the course of murine Mtb infection, the chronic activation of ESAT-6-specific CD4^+^ T lymphocytes induces terminal differentiation of these cells, which exhibit phenotypic and functional signals of exhaustion (Moguche et al., 2017). Terminally differentiated exhausted T cells are more susceptible to undergo apoptosis upon subsequent antigen stimulation (Saeidi et al., 2018), and in this case, it is possible that additional stimulation provided by ESAT-6 peptide infusion may have induced apoptosis in these cells, therefore reducing their numbers in the lungs.

In order to formally test if an increase in the levels of ESAT-6 specific Th1 cells could enhance the control of bacterial replication by the host and accelerate its clearance by antibiotic therapy, we performed sequential adoptive transfers of a Th1 polarized ESAT-6-specific CD4^+^ T cell clone (C7 cells) to Mtb-infected mice in the presence or absence of antibiotic treatment over a period of 4 weeks (**Figure 2P**). Transferred cells were detected in the lungs of recipient mice at the experiment’s endpoint and represented a higher proportion of the total pool of pulmonary activated CD4^+^ T cells in antibiotic-treated animals than in untreated mice (**Figures 2R, S**). Animals treated with C7 cells had similar bacterial loads compared to untreated mice. Animals treated with antibiotics or antibiotics + C7 also had similar pulmonary bacterial loads, although both groups presented reduced numbers of bacilli compared to untreated mice or mice receiving C7 cells alone (**Figure 2Q**). We hypothesized above that the reduction of ESAT-6-specific CD4^+^ T lymphocytes in antibiotic treated mice may be a reflection of decreased expression of ESAT-6 by bacteria in response to antibiotic-induced stress. Such scenario would explain the failure of C7 Th1 cell infusion strategy in reducing bacterial loads in antibiotic treated Mtb-infected mice, because in that event, the increase in the frequency of ESAT-6 specific Th1 cells would be useless, since there would be reduced levels of MHC-II associated ESAT-6 peptides on the surface of infected cells, thereby limiting the response of these lymphocytes.

We had previously observed (**Figure 1**) that contrary to what was found regarding ESAT-6-specific Th1 responses, antibiotic treatment induces an increase in the frequency of Ag85B-specific CD4^+^ T lymphocytes along with higher IFN-γ and TNF production by these cells. We therefore performed parallel experiments using Ag85B peptide treatment (**Figure 2I**) as well as sequential adoptive transfers of a Th1 polarized Ag85B-specific CD4^+^ T cell clone (P25 cells) (**Figure 2P**) to test if further enhancement of this response, in contrast to the ESAT-6 response, could help restrict bacterial growth or accelerate its clearance by antibiotics. Similar to our results with ESAT 6, we again found that bacterial loads recovered from lungs of Ag85B-treated and untreated mice were similar. Mice treated with antibiotics or antibiotics + Ag85B presented lower numbers of bacilli in lungs compared to untreated or Ag85B-treated mice, however, no significant difference was found between the bacterial loads of antibiotics and antibiotics + Ag85B-treated mice (**Figure 2M**). In contrast to the results obtained with ESAT-6 treatment, mice receiving Ag85B alone displayed a major increase in the frequency of IFN-γ and TNF double producer pulmonary CD4^+^ T cells in response to Ag85B peptide stimulation compared to untreated mice. However, no difference was observed between untreated, antibiotics and antibiotics + Ag85B-treated mice groups which all presented lower frequencies compared to animals treated with Ag85B-treated alone (**Figures 2N, O**). Importantly, the increase in IFN-γ^+^ TNF^+^ CD4^+^ T cells in Ag85B-treated mice was not accompanied by changes in pulmonary bacterial loads (**Figure 2M**).

The enhancement in IFN-γ and TNF production by antigen-specific CD4^+^ T lymphocytes following Ag85B peptide administration was previously observed and this strategy was also found to promote improved control of bacterial replication in Mtb infected mice (Bold et al., 2011; Moguche et al., 2017). This was not observed in our experiments. However, in the studies cited above, the authors did not perform Ag85B peptide administration in conjunction with antibiotics. In contrast, two additional studies demonstrated that the administration of an Ag85A DNA vaccine in combination with antibiotic therapy was effective in preventing the reactivation of Mtb infection after cessation of treatment, which correlated with heightened IFN-γ production in response to the antigen in mice that received DNA vaccination in conjunction with antibiotic therapy (Ha et al., 2003; Ha et al., 2005). Therefore, although our results did not indicate a beneficial effect of Ag85B administration in accelerating bacterial clearance in the first month of therapy, the studies above suggest that this effect may be achieved at later time points, perhaps because the CD4^+^ T cell-specific responses to Ag85 proteins, in contrast to those to ESAT-6, can be boosted during the chronic phases of Mtb infection (Moguche et al., 2017).

In the single experiment in which we performed adoptive transfer of Th1-polarized P25 cells (**Figure 2P**), we also detected a higher frequency of transferred cells in the total pool of pulmonary activated (CD44^+^) CD4^+^ T cells in RHZ-treated animals compared to untreated mice (**Figures 2U, V**). However, as observed for C7 cell transfer, P25 cell transfer did not result in changes in pulmonary bacterial burden. Also, mice treated with RHZ or RHZ + P25 cells had lower bacterial loads compared to untreated animals and Ag85B-treated mice, while no difference was found between the RHZ and RHZ + P25-treated groups (**Figure 2T**).

Thus, these attempts at accelerating pathogen clearance during the initial month of antibiotic therapy using approaches designed to enhance the magnitude of Th1 responses, and in particular those specific to ESAT-6 and Ag85B were uniformly unsuccessful. However, our results do not exclude the possibility that such approaches might have an impact at later time points after treatment initiation, especially if continued for longer periods, aspects that were not assessed in the experiments presented here. Also, there is a possibility that they may have a beneficial effect if combined with a suboptimal antibiotic regimen, such as the administration of a single or a combination of only two antibiotics, as observed in many studies on host-directed therapies to TB. Nonetheless, the use of suboptimal antibiotic dosage would be an unlikely scenario in the treatment of TB patients. Although largely negative, our findings argue that the reduction in Th1 response seen following antibiotic treatment is not a critical factor limiting the therapeutic efficacy of the drug regimen.

### Further Considerations on Host-Directed Therapies Focused on Enhancing CD4^+^ T Cell Reactivity to *M. tuberculosis* Antigens

The results presented here suggest that pulmonary IL-12 production and Th1 immunity against ESAT-6 and Ag85B may not be appropriate targets for host-directed therapeutic intervention against pulmonary TB. However, there are other studies in which similar strategies were used that resulted in increased resistance to Mtb and that when used adjunctively improved antibiotic therapeutic outcomes. These are discussed below.

IL-12 has been previously tested as an adjuvant for antibiotic therapy in a patient with disseminated TB who did not respond to conventional antibiotic therapy, although no drug-resistant bacteria were detected during the whole duration of treatment. The adjunctive therapy with IL-12 was associated with improvement in the patient’s clinical manifestations and better containment of the previously uncontrolled bacterial dissemination, however, the patient still relapsed and required 5 extra months of therapy (Greinert et al., 2001). Therefore, although we found no improvement in the control of pulmonary bacterial loads upon IL-12 administration, the results of the above-mentioned study suggest that the cytokine may improve the effects of antibiotic treatment in controlling Mtb replication in extrapulmonary sites. In addition, our results do not exclude the possibility that strategies able to sustain or enhance pulmonary IL-12 levels in a controlled manner may be beneficial when performed in conjunction with antibiotic therapy. As mentioned previously, two studies by Elías-López et al. and Mata-Espinosa et al., in which IL-12 was supplemented orally in food or *via* the airways using an adenoviral vector expressing the cytokine promoted beneficial outcomes during Mtb infection (Elias-Lopez et al., 2008; Mata-Espinosa et al., 2019), although the authors did not test these approaches in conjunction with antibiotic therapy. The strategies utilized for IL-12 supplementation in these studies may have prevented the detrimental side effects associated with the accumulation of exacerbated systemic levels of the cytokine, therefore explaining its beneficial outcome during experimental TB. Also, distinct from the experimental model used by us, in both studies performed, IL-12 was provided at different time points post Mtb-infection and employed high dose challenge of BALB/c mice, an inbred strain which usually produces lower levels than C57BL/6 animals of IFN-γ (Arko-Mensah et al., 2009), a cytokine that plays a critical role in the detrimental effects of IL-12 supplementation (Car et al., 1995; Ryffel, 1997).

A critical aspect to be considered when developing strategies for enhancing the magnitude of the CD4^+^ T cell responses against Mtb during antibiotic treatment is the choice of the right antigens/epitopes. We and other authors have used widely characterized Mtb antigens that are known to be highly immunogenic, such as those from Ag85 proteins and ESAT-6 (Brodin et al., 2004; Kroesen et al., 2019). These antigens are expressed by actively replicating bacilli, which are the first to be eliminated by antibiotic treatment. However, after the first rounds of therapy, slowly and non-replicating/latent bacilli persist, which have an extremely low metabolic activity and express a different set of antigens compared to actively dividing bacteria. The expression of many of these antigens is induced in response to the stress caused by immune response and chemotherapy and the non-replicating bacilli are known to be less vulnerable to the action of antibiotics (Baer et al., 2015). In fact, the persistence of non-replicating bacteria is intimately associated with the need for the extremely lengthy therapeutic regimen that is used in the standard treatment of Mtb infection (Connolly et al., 2007).

A better strategy to be employed during antibiotic therapy, might be to amplify CD4^+^ T cell responses against antigens selectively expressed by these non-replicating bacteria. A recent study found that isoniazid treatment induces the expression of a stress-responsive enzyme by Mtb, which results in increased levels of CD4^+^ T cells specific for this protein in antibiotic treated mice. The authors also found that therapeutic vaccination with a DNA vaccine for this enzyme results in improved bacterial clearance in mice treated with isoniazid (Chuang et al., 2020). RUTI is a vaccine candidate composed of fragments of Mtb bacilli that were grown in culture conditions that mimic immune response-driven stress, which result in heightened expression of antigens that are present in non-replicating bacilli (Cardona, 2006). The administration of RUTI vaccine during or after short term antibiotic treatment in experimental Mtb infection resulted in better control of bacterial reactivation (Cardona et al., 2005; Guirado et al., 2008). ID93 is another candidate vaccine for TB, which is composed of three Mtb virulence-associated antigens and one Mtb latency-associated antigen. This vaccine is formulated with the GLA-SE synthetic adjuvant, a TLR4 agonist, and has been considered safe and immunogenic in phase 1 and 2a clinical trial studies in humans (Penn-Nicholson et al., 2018; Day et al., 2020). When administered to Mtb-infected mice in conjunction with antibiotics, ID93 + GLA-SE was able to improve bacterial clearance and reduce relapse compared to antibiotic treatment alone (Coler et al., 2013). Importantly, in all of the above-mentioned studies, the beneficial outcomes were associated with enhanced CD4^+^ T cell responses towards the bacterial antigens.

A final aspect that needs to be considered in the development of all of the strategies mentioned previously, is the route of administration. Several candidates for immunization protocols, either for preventive or therapeutic purposes, were shown to induce strong Th1 systemic responses against several Mtb antigens when administered by the intramuscular route (Scriba et al., 2020). However, it is important that these cells be able to migrate to the lesion sites in the pulmonary parenchyma and activate infected phagocytes to eliminate the internalized bacteria (Sakai et al., 2014). In this regard, it has been demonstrated that the delivery of the immunizing agent by either mucosal (Wang et al., 2004), and as recently shown, by intravenous route (Darrah et al., 2020), can induce enhanced migration of memory and effector cells to the pulmonary tissue compared to the conventional intramuscular route, resulting in improved protection against infection. For this reason, further studies evaluating different routes of therapeutic vaccination in the context of antibiotic treatment are needed.

In summary, the enhancement of CD4^+^ T cell function as a strategy of host-directed therapy during antibiotic treatment in tuberculosis has a great potential in the development of more rapid and effective TB therapies. However, there are still important aspects that require further research before the best strategies can be defined. In particular a better understanding of the host-pathogen interaction during antibiotic treatment is needed. This involves a better characterization of the changes in the dynamics of the CD4^+^ T cell responses to Mtb antigens and alterations in bacterial and infected host cell gene expression and metabolism that take place during antibiotic treatment to TB, in either fast, slow or poor responding hosts.

## Data Availability Statement

The raw data supporting the conclusions of this article will be made available by the authors, without undue reservation.

## Ethics Statement

The animal study was reviewed and approved by NIAID Animal Care and Use Committee (ACUC).

## Author Contributions

Conceptualization: DC and AS. Experimental design: DC, EA, BA, and AS. Investigation: DC, EA, SN, LM, and BA. Data analysis and interpretation: DC, EA, SN, BA, and AS. Resources: AS. Writing: original draft - DC. Review and editing: DC, EA, SN, BA, and AS. Supervision: AS. Funding acquisition: AS. All authors contributed to the article and approved the submitted version.

## Funding

This work was funded by the Intramural Research Program of the NIAID. DC is currently funded by Fundação de Amparo à Pesquisa do Estado de São Paulo (FAPESP), grant #2019/08445-8. SN, EA, and AS are funded by the Intramural Research Program of the NIAID, NIH. BA is funded by the Intramural Research Program of the Fundação Oswaldo Cruz, Brazil, and is a senior fellow of the Conselho Nacional de Desenvolvimento Científico e Tecnológico, Brazil.

## Conflict of Interest

The authors declare that the research was conducted in the absence of any commercial or financial relationships that could be construed as a potential conflict of interest.
